# A Systematic Investigation into Aging Related Genes in Brain and Their Relationship with Alzheimer’s Disease

**DOI:** 10.1371/journal.pone.0150624

**Published:** 2016-03-03

**Authors:** Guofeng Meng, Xiaoyan Zhong, Hongkang Mei

**Affiliations:** 1 Computational Modeling Sciences, Platform Technologies and Science, GlaxoSmithKline Research & Development, Shanghai, China; 2 Neurodegeneration DPU, GlaxoSmithKline Research & Development, Shanghai, China; The Florey Institute of Neuroscience and Mental Health, AUSTRALIA

## Abstract

Aging, as a complex biological process, is accompanied by the accumulation of functional loses at different levels, which makes age to be the biggest risk factor to many neurological diseases. Even following decades of investigation, the process of aging is still far from being fully understood, especially at a systematic level. In this study, we identified aging related genes in brain by collecting the ones with sustained and consistent gene expression or DNA methylation changes in the aging process. Functional analysis with Gene Ontology to these genes suggested transcriptional regulators to be the most affected genes in the aging process. Transcription regulation analysis found some transcription factors, especially Specificity Protein 1 (SP1), to play important roles in regulating aging related gene expression. Module-based functional analysis indicated these genes to be associated with many well-known aging related pathways, supporting the validity of our approach to select aging related genes. Finally, we investigated the roles of aging related genes on Alzheimer’s Disease (AD). We found that aging and AD related genes both involved some common pathways, which provided a possible explanation why aging made the brain more vulnerable to Alzheimer’s Disease.

## Introduction

Aging is a natural biological process for all the species. It is associated with degenerative loss of tissue or cellular functions in the brain [1, 2], which increases the risk for neurological diseases, such as Parkinson’s Disease (PD), Alzheimer’s Disease (AD) and many other conditions [3]. With the increasing percentage of older people in the population and the resulting huge expenditure on age related diseases [4], studies on aging and these related diseases have drawn increasing attention.

Aging is viewed as a complex biological process [5]. Many reviews have summarized the factors that regulate the aging process [6–9]. In GenAge database [10], 298 genes have been collected from published works for their relationship with aging. Even with such knowledge, the mechanisms of aging are still far from fully understood. On one hand, aging is a progressive process and it is a considerable challenge to study the dynamic changes that occur during a complete lifespan in human. On the other hand, aging is a synergistic process resulting from the combined effects of a series of biological alterations [2]. It is impossible to fully recover the mechanism of the aging process by manipulation to few genes. These challenges make it necessary to perform systematic investigations at a genomic level.

Using high throughput gene expression or DNA methylation data, an opportunity for systematic investigation exists at the transcriptomics and epigenetics level. Many studies have reported effects of aging on DNA methylation and gene expression [11, 12] and even successfully used that information to predict the age of individuals [13, 14]. Those studies support the idea that DNA methylation and gene expression play important roles in the aging process. Systematic investigation of aging related gene expression or DNA methylation will help to uncover the synergistic effects of different regulatory mechanisms and is also useful for finding the connections between aging and age related diseases [15].

In this work, we define aging related genes as the genes with sustained and consistent changes occurring in the aging process. We identify these genes in the brain by selecting the ones that have expression or DNA methylation profiles, which correlate with the age of healthy people. Functional annotation of these genes suggests that transcription factors are the most affected genes in the aging process. Thus, we studied their regulation by enrichment analysis using motifs and literature reports. Transcription factor Specificity Protein 1 (SP1), which also has an age-correlated expression profile, is suggested to take regulatory roles for aging related genes. To further understand the function of aging related genes, we performed module-based functional analysis and confirmed these genes to be involved in aging related biological processes or pathways. Finally, aging related genes were investigated for their relationship with Alzheimer’s Disease (AD). We found both aging and AD related genes to be involved in the same pathways and the shared pathways were supported by published works to associate with both AD and the aging process. Our results suggest that aging and AD involve alterations to the same biological processes and that the progress of aging makes the brain more vulnerable to Alzheimer’s Disease.

## Materials and Methods

### Aging related genes in brain

Genes with sustained and consistent changes in the aging process, are supposed to be aging related genes. To find such genes in brain, we collected two sets of microarray data: GSE15745 [16] and GSE30272 [17], in which 147 and 269 healthy participants were measured for both gene expression and DNA methylation. In both data sets, the age of participants covered a long life span from young (15 years) to old age (101 years). The normalized data were downloaded from GEO database (http://www.ncbi.nlm.nih.gov/geo/). The quality of microarray data was evaluated by hierarchical clustering and principal component analysis to remove outliers. Three vectors were constructed, which respectively recorded the information of normalized gene expression, the beta values of the DNA methylation and the age of the participants. Then gene expression and DNA methylation were analyzed for their profile similarity with age using Spearman’s correlation.

To determine the correlation cutoff, we performed shuffling by assigning age to random samples and new correlations were calculated. In each iteration of simulation, we found the minimum and maximum correlation values, which defined the range of random correlations. After 1000 rounds of shuffling, the correlation cutoff was determined by choosing a range so that more than 95% of random correlation were within such a range. We also transformed the random correlation by Fisher’s transformation and calculated their means and standard deviations. *Z*-score based *P*-value was used to describe the significance of the selected correlation cutoff. In this process, positive and negative correlations were both considered.

### Enrichment analysis

We performed enrichment analysis to find the regulators or functional annotation for a group of co-expressed genes. For *k* input genes, the number of genes with functional annotation or regulators is *x*. For *n* whole genomic genes, the number of genes with functional annotation or regulators is *p*. We used Fisher’s exact test to evaluate if the observed *x* genes resulted from random occurrences. We used the following R codes to calculate the p-value:

> m = matrix(c(x, k-x, p-x, n-k), ncol = 2, byrow = T)
> p = fisher.test(m, alternative = “greater”)$p.value

### Transcription regulation analysis

Based on an assumption that the co-expressed genes are co-regulated, we performed transcriptional regulation analysis to predict the regulators for co-expressed genes. In this analysis, two strategies were applied: (1) motif-based over-representation analysis; (2) literature reports-based enrichment analysis.

Motif-based over-representation analysis (ORA) is used to recover the enriched regulatory motifs from a group of co-expressed genes. We used oPOSSUM (Version 3.0) [18] as the implementation. In our previous work [19], we have evaluated the performance of oPOSSUM with differentially expressed genes from transcription factor manipulated microarray experiments and optimized parameter setting of oPOSSUM as following: promoter length (from −2000 to +2000 bp), sequence conservation (top 30%), input gene number (200). In a case, when the input gene number exceeded 200, we ranked genes based on their correlation with age and chose the top 200 ones for ORA. The enriched transcription factor (TF) binding motifs were selected at a cutoff of *z* > 20 and Fisher’s *p* < 10.

Literature reports about TF-target regulation were also used for enrichment analysis. In this step, the annotated transcription regulation including “transcription regulation” and “influence gene expression”, were selected from MetaBase (http://thomsonreuters.com/metabase/). The input genes were evaluated for the enrichment of annotated regulators. If any regulator was significantly enriched, it would be predicted to regulate the expression of the input genes.

To find the gene-specific regulators, we applied the method CSTP [20] to construct a transcriptional regulatory network. The assumption of this analysis is that the regulators of one gene can be recovered by analysis to the genes with similar expression profiles. In this step, gene expression data of all samples were used as input matrix for CSTP. All the aging related transcription factor genes were predicted for their transcription regulators. As results, a regulatory network could be constructed by connecting the predicted regulators and their targets.

### Gene functional analysis with Gene Ontology and pathway annotation

We performed Gene Ontology (GO) enrichment analysis using David [21] to computationally annotate the functions of a group of genes. Using the genomic genes as the background, genes was evaluated for enrichment of GO biological process terms. If a significant *p*-value was observed (e.g. *p* < 0.01), the genes were considered to be related to the enriched biological process.

The same functional analysis could be performed using pathway genes. We collected the pathway genes by combining the pathway annotation from Metabase (http://thomsonreuters.com/metabase), GenMapP (http://genmapp.org/), Biocarta (www.biocarta.com) and Gene Ontology. For a list of input genes, we evaluate their enrichment of pathway genes using Fisher’s exact test. If significant *p*-values were observed, pathways were assumed to regulate the input genes.

### Functional module analysis to aging genes

The assumption for this analysis is that genes with similar expression profiles involve in the same pathways or biological processes. We clustered the aging related genes into different modules based on the similarity of their gene expression profiles. The functional annotation of each module was performed by enrichment analysis to Gene Ontology annotations. The gene expression similarities of gene pairs were described by the correlation distance (1-*r*) across all the samples. R package WGCNA [22] was used to find the gene modules. We displayed the module genes using hierarchical clustering with different colors to label the memberships to the modules. The functional and pathway enrichment analyses were performed for each of the modules.

### Alzheimer’s disease (AD) related genes

AD related genes were collected in two ways. Firstly, expression experiments were identified where the AD patients and controls were both measured for genomic genes. In this step, we found three microarray experiments with good quality: GSE5281 [23], GSE26972 [24], GSE48350 [25]. After removing the irrelevant samples e.g. samples with young age, we performed differential expression analysis and utilized meta-analysis to find genes shared by these three experiments. Another method was from text mining of the published works. In this process, the annotation for AD from IPA (http://www.ingenuity.com/products/ipa) and DisGeNet (www.disgenet.org/) were used. We filtered the low-confident genes by manually removing the ones with only evidences of expression or those not supported by independent evidences.

## Results

### Aging related genes in brain

**Fig 1:**
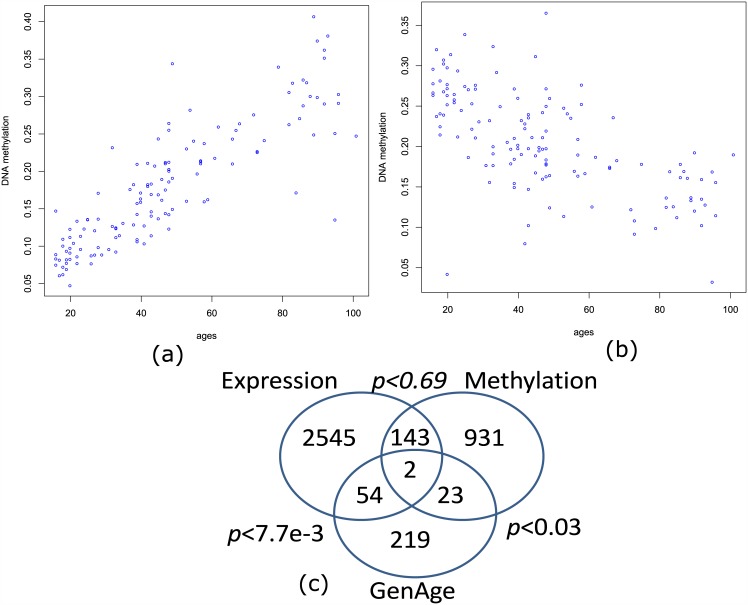
Aging related genes and DNA methylation sites. (a)RUNX3, positively correlated with age; (b)C10orf10, negatively correlated with age; (c) overlap of genes with aging related expression and DNA methylation and genes from the GenAge database.

We defined the aging related genes as the ones with sustained and consistent expression or DNA methylation changes in the aging process. To find such genes, we calculated Spearman’s correlation between DNA methylation or gene expression and age. Based on simulation results, we used |r| > 0.3 as the cutoff to select aging related DNA methylation sites and genes. Analysis of GSE15745 [16] suggested genes or sites to have age-correlated profiles in four brain regions (see Table 1). Taking the frontal cortex (FCTX) as an example, we observed 2097 positively and 276 negatively correlated DNA methylation sites. The maximum positive correlation *r* = 0.85 was observed with a site located in the upstream CpG island of RUNX3 gene (see Fig 1(a)). On the contrary, the minimum correlation *r* = −0.63 was observed with C10orf10 gene (see Fig 1(b)). A similar analysis using expression data also suggested genes to have aging related profiles (see Table 1). Considering the conservation of the aging process [26, 27], we assumed the aging related genes to be shared by different brain regions. Therefore, we collected aging related DNA methylation sites and genes by checking their overlaps across four brain regions. We observed 1243 DNA methylation sites and 2743 genes to be shared by at least three regions at a cutoff of |r| > 0.3 (see Table 1 and S1 Table). These genes were defined as aging related genes and they were used for next analysis step.

**Table 1:** The aging associated genes and DNA methylation sites.

Type	Regions	*r* > 0.3	*r* < −0.3
DNA methylation sites	CRBLM	150	367
	FCTX	2097	276
	PONS	2091	1539
	TCTX	3347	535
	> 3 Regions	1067	176
Expressed genes	CRBLM	2090	2549
	FCTX	2065	2906
	PONS	2828	1815
	TCTX	2075	2122
	> 3 Regions	1265	1478

CRBLM: cerebellum; FCTX: frontal cortex; PONS: pons; TCTX: temporal cortex

Next, we evaluated the reliability of aging related genes by performing the same analyses in an independent experiment GSE30272 [17]. The results from two data sets were compared and the overlapping results were showed in S1 Fig. In the FCTX region, we found 1297 DNA methylation sites, about > 50% of aging related sites in GSE15745, to be observed in GSE30272 (*p* < 2.2*e* − 16). The same comparison was performed for genes with aging related expression profiles. 474 genes, about 23.4% of total aging related genes, were shared by two datasets with a significance at *p* < 0.01. This result indicated that both aging related DNA methylation and gene expression had consistent profiles in independent experiments. However, more overlap was observed with DNA methylation, which suggested DNA methylation profiles to be more stable in the aging process.

In the GenAge database [10], 298 aging related genes have been identified from published works. Different from our definition, those genes were determined by manipulation experiments in model animals or by association studies in human. In Fig 1(c), we show the overlap of GenAge genes with our genes. We observed 56 GenAge genes to have aging related expression profiles and 25 genes to have aging related DNA methylation profiles. Fisher’s exact test suggests shared genes to be significantly enriched at *p* < 0.0077 and *p* < 0.03, respectively.

Considering the roles of DNA methylation in gene expression regulation [28], we checked whether aging related DNA methylation was the causal reason for aging related gene expression. As shown in Fig 1(c), there are only 145 aging related genes having aging related DNA methylation sites, which covers about 5.2% of aging related genes. Fisher’s exact test fails to support this overlap to be significant at *p* < 0.05. This result suggests that DNA methylation is not the causal reason for aging related gene expression and there may be transcriptional regulation at other levels which involves the gene expression regulation in the aging process.

### Transcription regulatory genes are most affected in the aging process

We performed functional annotation of the aging related genes with David [21]. By mapping the aging related DNA methylation sites to nearby genes, 1099 aging related genes were selected. Analysis of the 1099 genes suggested “transcriptional regulation” to be the most enriched term. 153 genes, 13.9% of the 1099 input genes had transcription regulatory activity (*p* < 2.7*e* − 27) and 231 genes had DNA binding activities (p < 8.0*e*−14), which were also the most enriched term. The same analysis was performed for genes with aging related expression profiles. Among 1265 positively correlated genes, 158 genes were annotated to have transcription factor binding activity (*p* < 1.4*e* − 8), which was the most enriched term. However, these transcription related terms were not enriched with the negatively correlated genes. Overall, this analysis suggests that transcription regulators are the most affected genes at both DNA methylation and gene expression levels in the aging process.

### Transcription regulation of aging related genes

**Fig 2:**
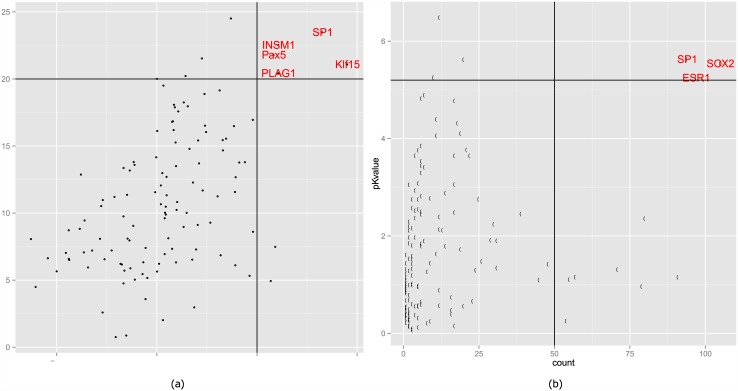
Enriched transcription factors using (a) the motif-based over-representation analysis and (b) literature reports-based enrichment analysis.

We performed motif-based over-representation analysis (ORA) to recover the enriched motifs for aging related genes. Taking genes from FCTX as an example, 5 TFs, including KLF15 (*p* < 7.9*e* − 22), SP1 (*p* < 3.2*e* − 24), INSM1 (*p* < 2.8*e* − 23), PLAG1 (*p* < 3.6*e* − 21) and PPARG (*p* < 3.1*e* − 8), were predicted to be significantly enriched among positively correlated genes (Fig 2(a)). To improve confidence, ORA was also performed in three other regions and found 7 TFs to be shared by at least three regions: KLF15, SP1, EGR1, IMSM1, ZFX, PLAG1 and GABPA. These TFs were potential regulators of aging related genes. Next, we checked the percentage of genes with predicted TF binding sites and found that, except for PLAG1, all the TFs bound to the promoters of more than 50% of input genes, which suggested that they were more likely to be regulators for the aging related genes. The same analysis was performed with negative correlated genes. However, we failed to find any motifs shared by at least three regions (see S2 Table).

Besides motif-based analysis, we also performed literature-reports based enrichment analysis. Three TFs were found to be significantly enriched, including SOX2 (*p* < 4.18*e* − 6), SP1 (*p* < 1.94*e* − 5) and ESR1 (*p* < 1.73*e* − 5) (see Fig 2(b)). These three TFs were also annotated to regulate more than 50% of aging related genes. Compared to the results from motif based enrichment analysis, different TFs were reported. However, SP1 was shared by both analyses, which further supports SP1 to be a regulator of aging related genes.

In the above analyses, we have predicted 9 TFs by motif and literature reports-based enrichment analysis. We also checked the gene expression profiles of predicted TFs in the aging process. We found SP1, KLF15, EGR1 and SOX2 to have aging related expression profiles at a cutoff of |r| > 0.3 (see Table 2). Expression of SP1, KLF15 and SOX2 were positively correlated while EGR1 was negatively correlated. Considering the fact that aging related genes were selected by choosing genes that had correlated profiles with age, we expected their regulators to have aging related profiles. Therefore, SP1, KLF15 and SOX2 and EGR1 were predicted to be more likely to be a transcriptional regulator of aging related genes.

**Table 2:** Expression correlation with age for 9 TF genes.

gene	FCTX	PONS	TCTX	CRBLM
KLF15	0.43	0.43	0.42	0.51
SP1	0.40	0.39	0.39	0.39
EGR1	−0.57	−0.45	−0.57	−0.41
INSM1	0.18	0.22	0.23	−0.13
ZFX	−0.11	−0.25	−0.18	−0.16
PLAG1	−0.11	0.29	−0.06	0.51
GABPA	−0.12	−0.16	−0.26	−0.26
SOX2	0.31	0.36	0.31	0.31
ESR1	−0.14	0.02	−0.13	0.001

To validate the results of enrichment analysis, we used the SP1 binding sites determined by ChIP-sep from ENCODE Project (http://www.genome.gov/encode/) and checked their enrichment in the promoter regions of aging related genes. Among 2743 aging related genes, we found 60.2% of them to be associated with at least one SP1 binding site at their promoter region in a range from −2000bp to 2000bp. This observation was further evaluated by randomly chosen genes. There were only 37.4% of random genes having SP1 binding sites and the significance for observed enrichment was *p* < 2.23*e* − 16. This observation confirms the results from enrichment analysis.

As the only transcription factor recovered by both motifs and literature reports-based enrichment analysis, SP1 has also been reported for its important roles in the aging process. In the GenAge database (http://genomics.senescence.info/), SP1 is annotated to be relevant to the human aging process based on evidence that links SP1 to the regulation of aging related genes. In Ryu et. al’s work [29], SP1 was reported to be an induced transcription factor in response to oxidative stress and the regulation of neuronal survival. In S.Y. Kim et. al’s work [30], SP1 was reported to be related to aging-dependent nucleocytoplasmic trafficking. In addition, Kumazaki et.al [31] reported SP1 to have altered TF binding activities in the aging process. SP1 has also been widely reported to be associated with aging by its involvement in the regulation of apoptosis [32–35].

### Regulator Network of transcription factors in aging process

**Fig 3:**
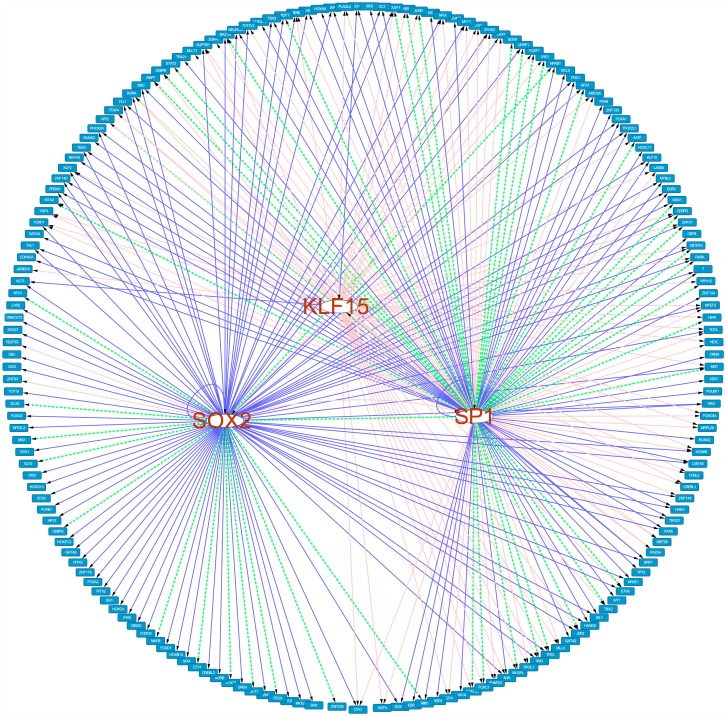
Transcription regulatory network of TF genes in aging process.

As presented in above section, transcription regulators are the most affected genes in the aging process. The complexity of transcription regulation makes it necessary to investigate their regulatory network. We applied a method, CSTP, to recover the transcription regulation for each of the aging related TF genes [20]. Using the gene expression data from GSE15745 [16], we found the co-expressed genes for each TF and the regulators of TF genes were recovered by enrichment analysis of the co-expressed genes with binding motifs and literature reports. The results were shown in S2 Fig. Motif enrichment analysis confirmed SP1 to be the most important regulator, which was predicted to regulate the greatest number of TF genes (68 out of 244 TF genes). Another enriched TF was KLF15, which regulated 62 TF genes. The enrichment analysis with literature reports suggested SOX2 and SP1 to be the key regulators, which regulated 105 and 94 TF genes, respectively. In the above section, we observed other significantly enriched TFs but they failed to be recovered by CSTP. By connecting SP1, KLF15 and SOX2 to their target genes, we constructed a regulatory network (see Fig 3). In this network, 179 TF genes were predicted with transcription regulators. Among them, 79 genes were regulated by only one of three TFs while 72 genes were regulated by two and 28 genes could be regulated by all three TFs. In this network, there were a total of 307 predicted regulations. Among them, 161 predicted regulations were observed both by enrichment to motifs and literature reports.

Next, we investigated the transcription regulation of SP1. CSTP suggested 8 TFs to regulate SP1 expression, including ESR1, RBPJ, WT1, NFKB1, FOXA1, RUNX3, IRF8 and ATOH1. However, none of them had strong correlation between their expression profiles and age. Therefore, these TFs were not supposed to be the only transcription regulators for SP1. Further investigation or more complex models is necessary to uncover the regulation of SP1.

### Functional Module analysis of the aging process

**Fig 4:**
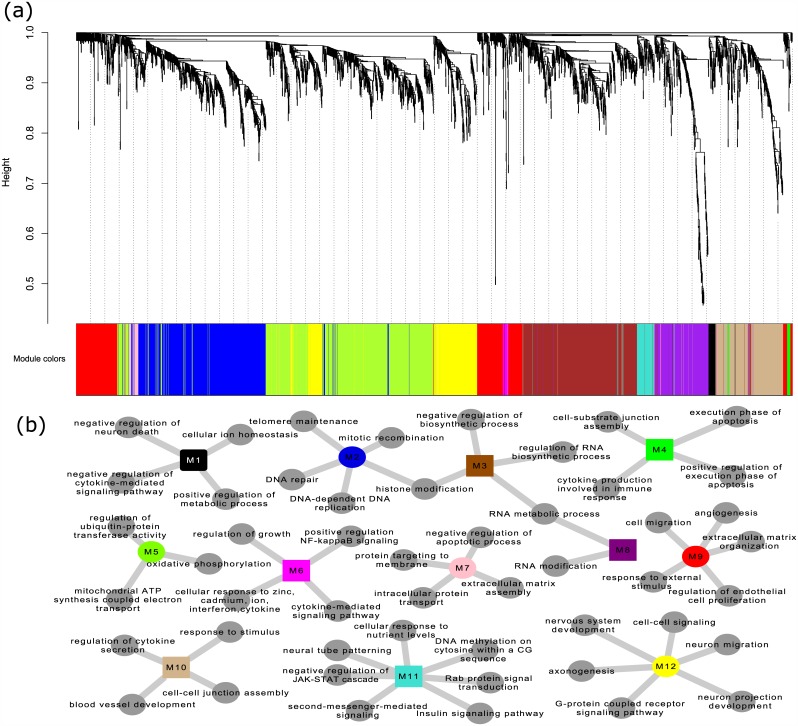
Network analysis of aging associated genes. (a)aging related genes were clustered into 12 modules based on their expression similarity; (b)each module was annotated for enriched pathways.

To fully understand the roles of the aging related genes, we performed functional module analysis with WGCNA [22]. Fig 4(a) shows the WGCNA analysis results. 2743 genes that had aging related expression profiles, were clustered into 12 modules (see S3 Table) and each module was computationally investigated for functional annotation.

As shown in Fig 4(b), 12 modules were predicted to involve different aging related biological processes. Aging is accompanied with accumulated cellular damage, especially to DNA [36]. In M2, we observed DNA repair-associated terms to be enriched. Cellular damage can block the cell cycle and cell proliferation [37, 38] (see M2, M6, M9, M11). Impaired functional loss and increasing vulnerability can result in apoptosis [39], which is a protective mechanism to avoid uncontrolled propagation of damaged cells [40] (see M4, M7). Many factors can lead to cellular damage. One important way is from the mitochondrial ATP synthesis process. The oxidative micro-environment of mitochondria leads to mtDNA damage and mitochondria deterioration [41] (see M5). Imbalance or loss of homeostasis and metabolism is an important feature of the aging process, which can lead to functional decline and increased risk of diseases [42–44] (see M1, M3, M8). Aging is characterized by dysregulated nutrient sensing [45]. In M1, we observed insulin signaling pathways to be enriched with aging related genes [46] (see M11). The roles of metabolic processes in the aging process can be confirmed by the relationship between longevity and calorie restriction [46]. Dysregulation of inflammatory senescent cells tends to lead to the secretion pre-inflammatory cytokines, which can affect many pathways and regulators in the aging process [47] (see M1, M4, M6, M10). The inter-cellular communication and cell-matrix interaction are widely reported to be involved in the aging process [48–50] (see M4, M7, M9, M10 and M12). Progressive loss of telomeres explains the limited proliferation ability of most cultured cells and it also is believed to be a causal mechanism of the aging process [51]. In M2, telomere maintenance associated terms are enriched. We observed many neuronal development associated terms, which may suggest that aging affects normal function of the neuron (see M1, M11, M12).

Overall, module analysis clustered the aging related genes into different modules and functional enrichment analysis indicated different module genes to be involved in different biological processes. By checking the published works, most of biological processes have been widely reported for association with the aging process. This result suggests that computational analysis to sustained and consistently changed genes can re-discover the known regulation of the aging process. This is also evidence for selected genes to be related to the aging process.

### Aging and Alzheimer’s disease

As one of diseases prevalent in older people, Alzheimer’s disease (AD) is generally believed to be related to the aging process. It is necessary to investigate the roles of aging related genes in the genesis of AD. We collected 1311 AD related genes by differential expression analysis of AD patients and 523 genes from published literature reports (see S4 Table). For the aging process, 3698 genes that had aging related expression or DNA methylation profiles, were selected as aging related genes (see S1 Table). We found 300 differentially expressed genes and 126 literature reported genes to be shared between AD and aging. Statistical evaluation with Fisher’s exact test suggested this overlap to be significant at *p* < 4.3*e* − 4 and *p* < 9.0*e* − 6 (see S2 Fig).

**Fig 5:**
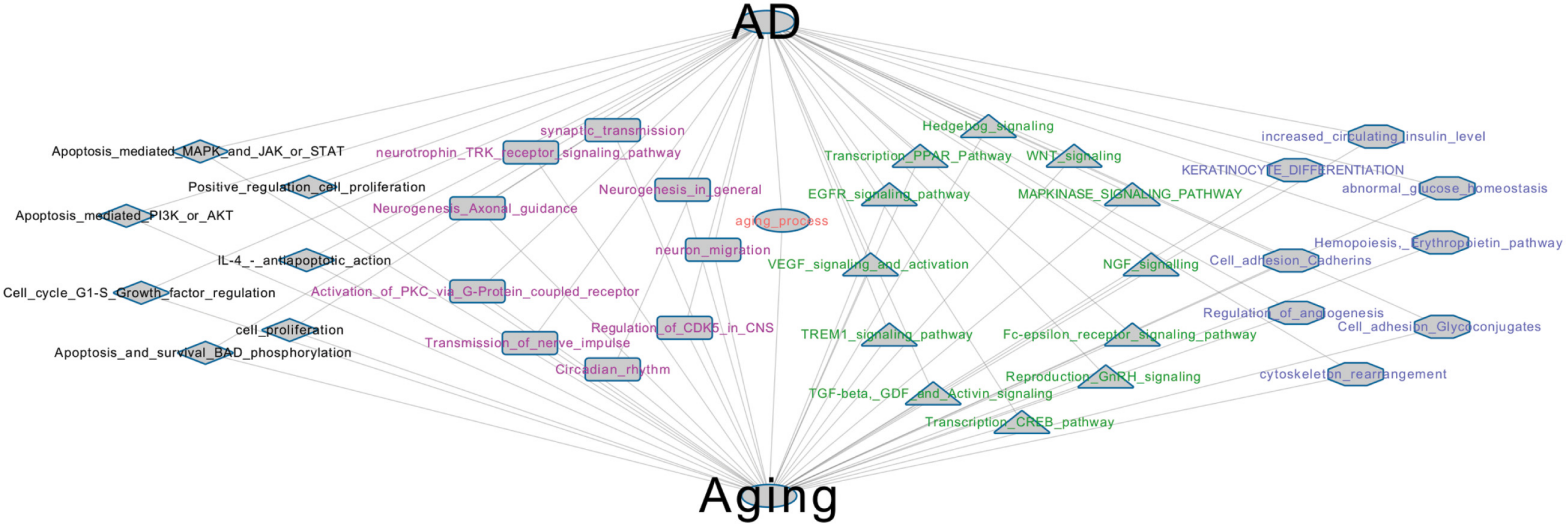
Pathway analysis for AD and the aging process. The shared pathways and processes from enrichment analysis of AD and aging related genes.

Next, we checked whether aging and AD related genes involved the same pathways. We applied a method introduced in previous work [52] and predicted the pathways enriched by both AD and aging related genes. Fig 5 showed the shared pathways. Based on functional similarity, the enriched pathways were clustered into four groups, which were tagged with four colors. In the black group (left 1), we observed pathways associated with apoptosis and cell proliferation to be enriched by both AD and the aging process. In AD, neuronal loss has been widely observed and reported [53]. Even though many reports suggest normal aging has less neuronal loss [54, 55], we observed cell death related terms to be enriched in aging related genes. Similarly, some pathways were observed to be enriched in the green group (left 3). Among them, the EGFR pathway [56], VEGF activation [57, 58], TGF-beta signaling pathway [59, 60], Hedgehog signaling pathway [61], Wnt signaling pathway [62], NGF signaling pathway [63] and GnRH signaling pathway [64] have been reported to be related to both AD and the aging process. From published reports collected in the ALZpathway database [65], it is found that most of these pathways have been annotated for their association with AD. In the blue group (left 4), we observed terms related to cellular interaction and metabolic processes. These two processes have been widely studied for their critical roles in both aging and AD [50, 66]. In the red group (left 2), there are terms associated with neuronal development and nerve impulse transmission. In AD and the aging process, brain aging is characterized by loss of neural circuits and plasticity, which is in line with the enrichment analysis results [67, 68].

Overall, we found pathways enriched by both AD and aging. Text mining analysis confirmed most of the shared pathways to be associated with both AD and aging. This result suggests that the aging genes are involved in the genesis of AD by affecting the AD-related pathways described above. This result also provides a possible explanation for why age is the biggest risk factor for age related neurological diseases.

## Discussion

In this work, we performed a computational study of the genes that had sustained and consistent expression or DNA methylation changes in the aging process of the brain. Our analyses confirmed these genes to be related to the aging process by involving many aging related pathways. Therefore, we called these genes as aging related genes, which could be either upstream regulators or downstream targets of the aging process. However, this is not exactly the same definitions used in many published works. For example, the genes from GenAge database are mostly determined by selecting ones where their experimental manipulation will delay or accelerate the aging process [10]. Other examples are from genome-wide differential expression analysis of the samples at different age [11, 69, 70]. The difference in definition mainly reflect the different emphasis for finding the aging related genes. The genes from this work give a new source for investigation to the aging process and age related neurological diseases.

Enrichment analysis of aging related genes suggested SP1 to be one of the most enriched TFs. One possible problem is the bias of the computational tool for some TFs, especially SP1. To remove this risk, we evaluated performance of oPOSSUM with randomly selected human genomic genes. We repeated it for 1000 iterations to check the distribution of z-scores and Fisher’s scores for all the TFs. The results are available in S3 Fig. We observed that the distribution of both z-scores and Fisher’s scores were within a small range, which did not exceed the significance threshold. This simulation result suggests that predicted TFs do not result from tool bias. Next, we checked whether oPOSSUM was powerful enough to recover the SP1 binding sites in real experimental data. We used one SP1 manipulated dataset from GSE37935 [71], where SP1 was the only manipulated regulator. Using the same parameter setting, we performed ORA to the differentially expressed genes and observed SP1 to be one of the most enriched TFs (see S3 Fig). This result suggests that SP1 can be recovered by ORA when it takes regulator roles in a biological process. Overall, our evaluation with simulated data and real data supports ORA to have good performance for both specificity and sensitivity.

With our analysss, we predicted some TFs, especially SP1, to transcriptionally regulate aging related genes. However, we observed relatively low correlation between SP1 expression and age. Therefore, it is unrealistic to expect SP1 or any other TFs to explain all the aging related gene expression. As observed in gene functional module analysis, aging related genes can be regulated by different TFs and pathways. To fully understand the transcription regulation of the aging process, systematic investigation with synergy of different TFs or pathways is imperative. This work only reveals limited information for the aging process and there is still a long way to understand the whole process.

## Supporting Information

S1 Fig Consistence of aging related expression and DNA methylation profiles in experiment replicates. The Aging related gene expression and DNA methylation profiles are compared between the results from two experiments: GSE15745 and GSE30272.
(PDF)

S2 Fig Overlap of aging related genes and AD related genes. The Aging related genes and AD related genes are compared for gene overlaps.
(PDF)

S3 Fig Evaluation of motif-based over-representation analysis. To check if there is any bias from over-representation analysis, we performed ORA to randomly chosen genes. This figure shows the results. We can see that the distribution of scores is within a small range, which does not exceed the significance threshold. Another analysis is over-representation analysis to GSE37935, where we found SP1 to be one of the significantly enriched TFs in the over-representation analysis.
(PDF)

S1 Table The list for aging related genes. The aging related genes were determined by finding genes with age-correlated gene expression and DNA methylation.
(XLSX)

S2 Table The results of enrichment analysis for negatively correlated aging related genes. The negatively correlated genes from four tissues were studied for transcriptional regulators using enrichment analysis.
(XLSX)

S3 Table Module genes in module analysis. The aging related genes are clustered into different modules where the colors indicate the membership in Fig 1.
(XLSX)

S4 Table Alzheimer’s disease related genes. The AD related genes are determined by differential expression analysis and text-mining annotation.
(XLSX)
