# Supplementary figures and images for: A Systematic Investigation into Aging Related Genes in Brain and Their Relationship with Alzheimer’s Disease
Source: PLoS One. 2016 Mar 3;11(3):e0150624. doi: 10.1371/journal.pone.0150624 (PMC4777381; doi:10.1371/journal.pone.0150624)

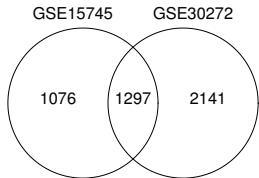

(a) DNA methylation sites

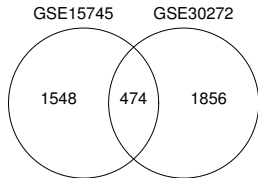

(b) Expressed genes

Supplement: S1 Fig — The Aging related gene expression and DNA methylation profiles are compared between the results from two experiments: GSE15745 and GSE30272. (PDF) [file pone.0150624.s001.pdf]

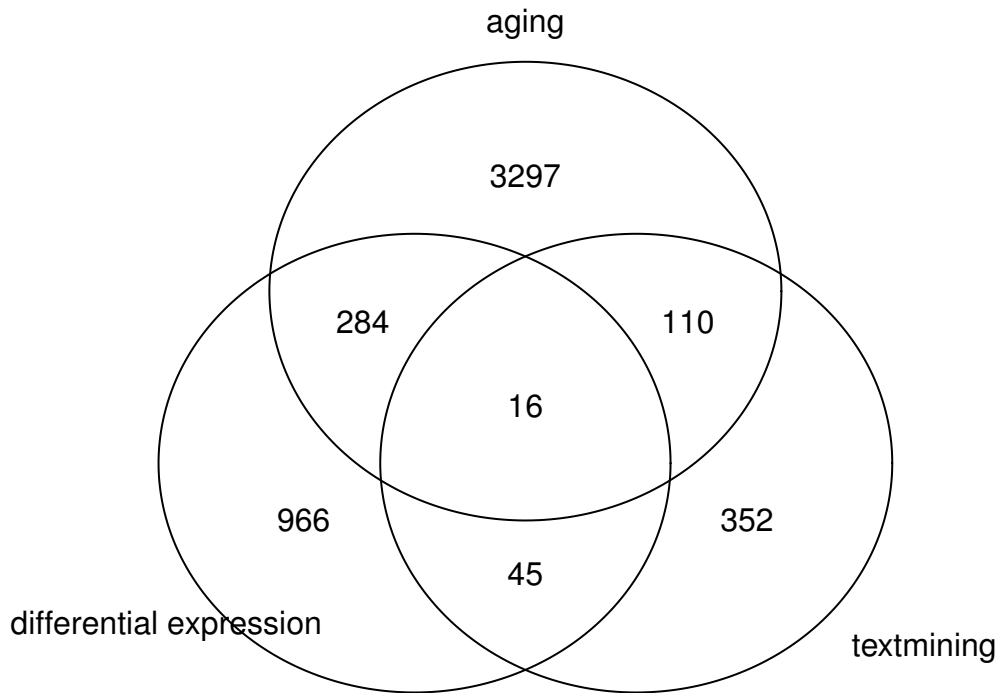

Supplement: S2 Fig — The Aging related genes and AD related genes are compared for gene overlaps. (PDF) [file pone.0150624.s002.pdf]

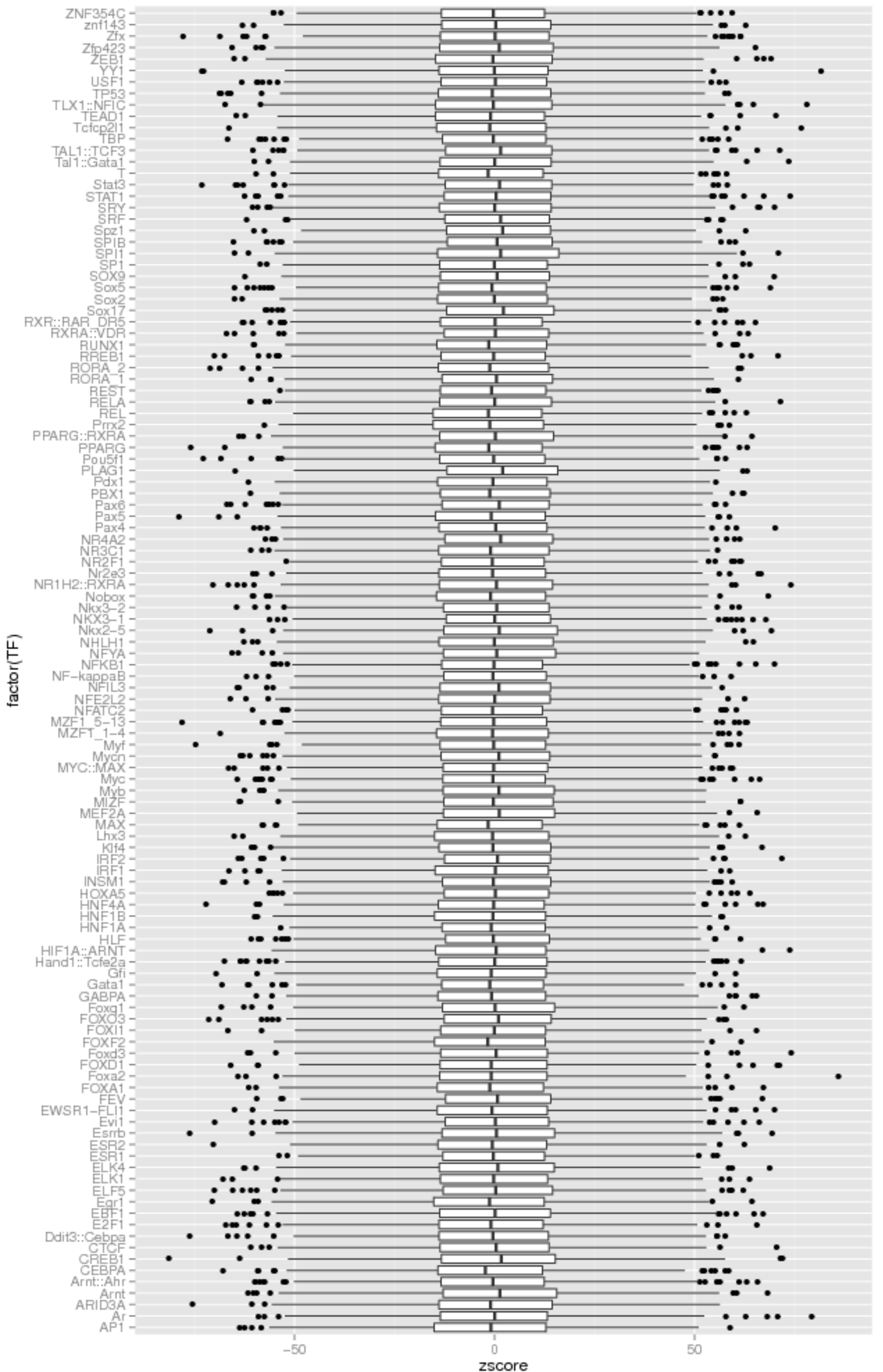

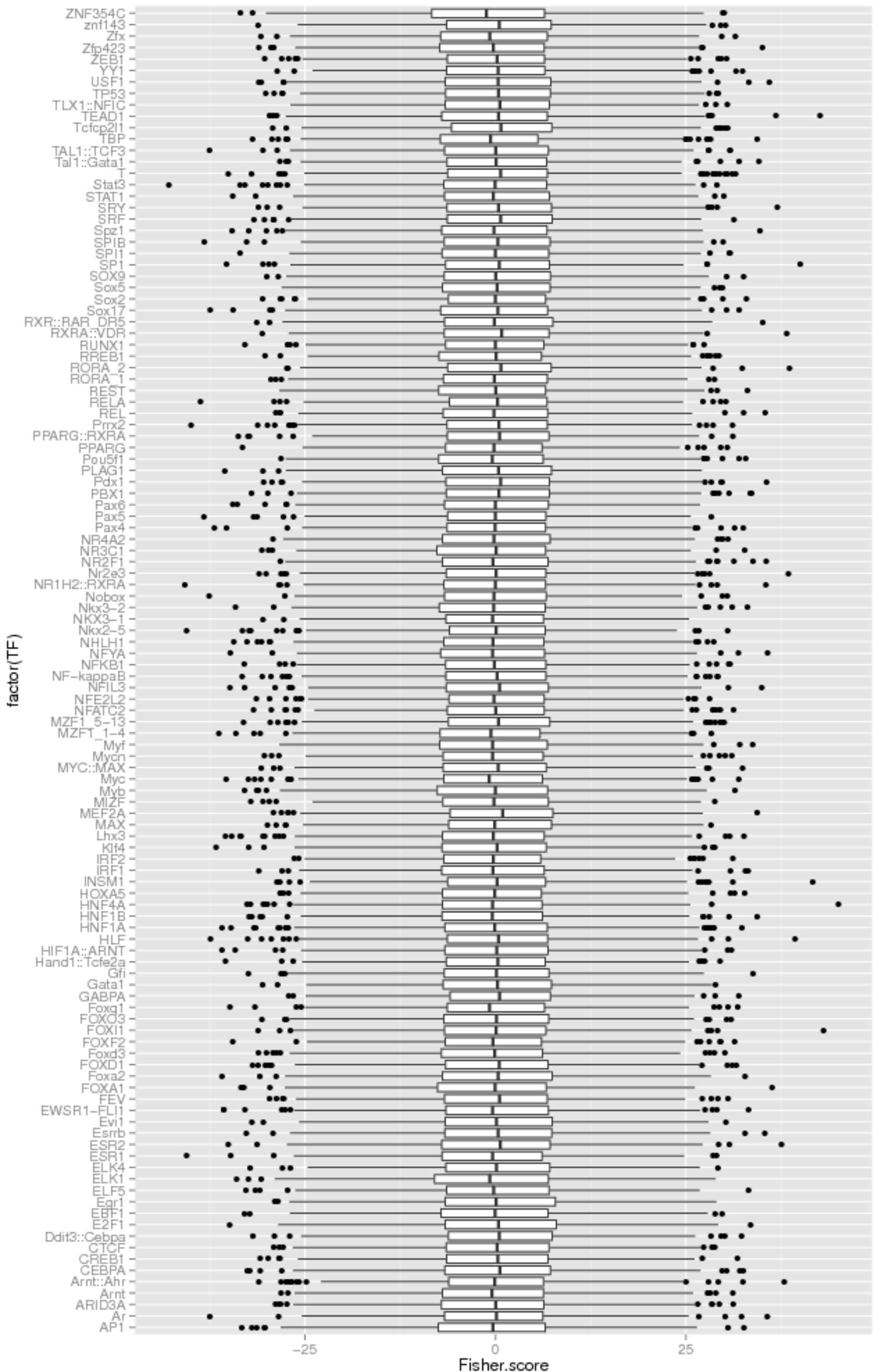

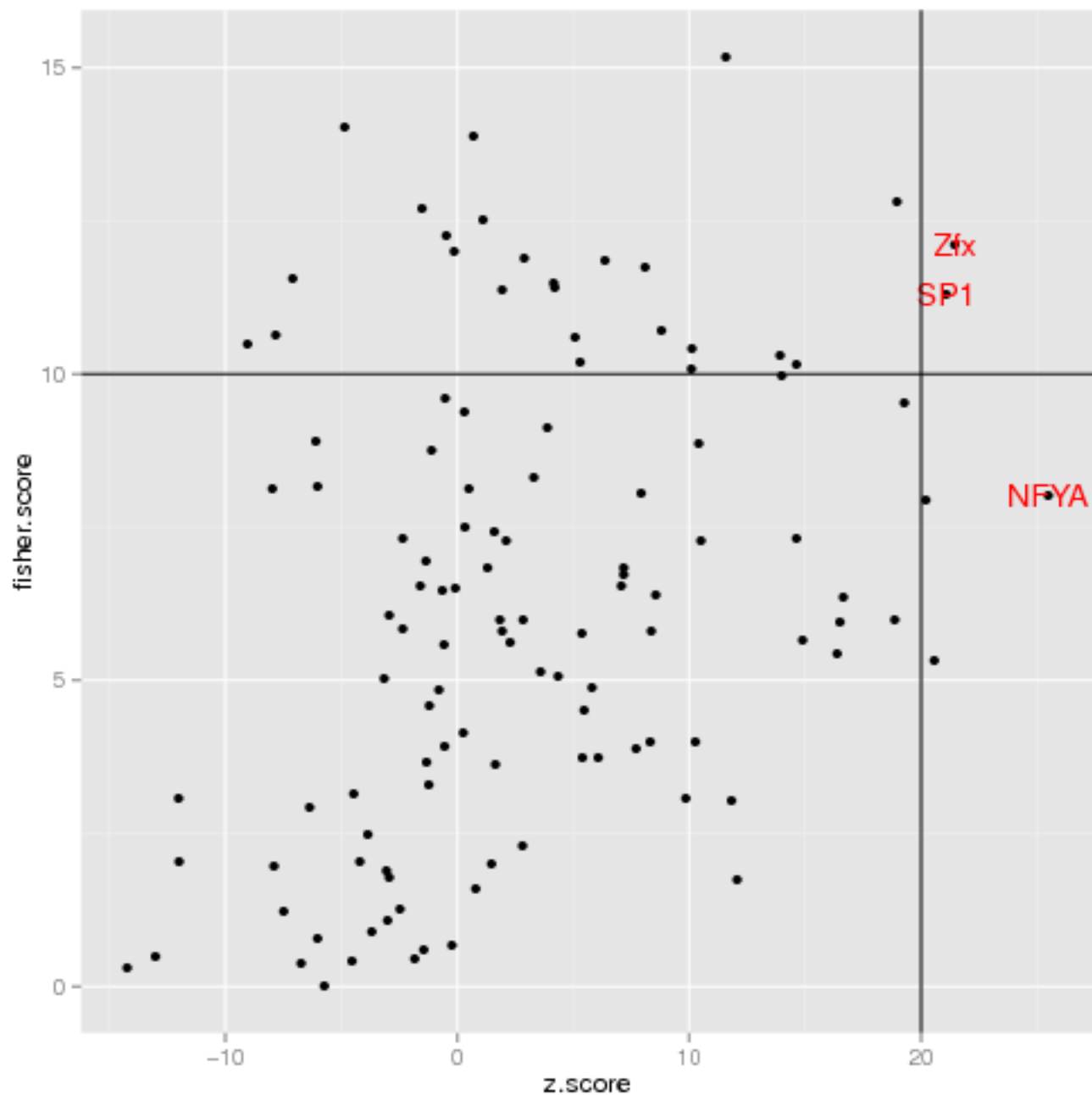

Supplement: S3 Fig — To check if there is any bias from over-representation analysis, we performed ORA to randomly chosen genes. This figure shows the results. We can see that the distribution of scores is within a small range, which does not exceed the significance threshold. Another analysis is over-representation analysis to GSE37935, where we found SP1 to be one of the significantly enriched TFs in the over-representation analysis. (PDF) [file pone.0150624.s003.pdf]
